# The C/EBPβ antagonist peptide lucicebtide (ST101) induces macrophage polarization toward a pro-inflammatory phenotype and enhances anti-tumor immune responses

**DOI:** 10.3389/fimmu.2025.1522699

**Published:** 2025-03-04

**Authors:** Claudio Scuoppo, Rick Ramirez, Siok F. Leong, Mark Koester, Zachary F. Mattes, Karen Mendelson, Julia Diehl, Franco Abbate, Erin Gallagher, Lila Ghamsari, Abi Vainstein-Haras, Gene Merutka, Barry J. Kappel, Jim A. Rotolo

**Affiliations:** Sapience Therapeutics, Inc., Tarrytown, NY, United States

**Keywords:** C/EBPβ, lucicebtide, tumor associate macrophages (TAM), M2-type macrophage, anti-pd 1 immunotherapy, ST101

## Abstract

Immune-checkpoint inhibitors (ICIs) have shown unprecedented success in a subset of immunogenic tumors, however a host of patients with advanced solid tumors fail to respond well or at all to immunotherapy. Refractory tumors commonly display a tumor microenvironment (TME) rich in immunosuppressive macrophages (M2-like) that suppress adaptive immunity and promote tumor progression. The ability to reprogram macrophages in the TME into an immune-active state holds great promise for enhancing responses to ICIs. Lucicebtide (previously referred to as ST101) is a peptide antagonist of the transcription factor C/EBPβ, a key activator of the transcriptional program in immunosuppressive macrophages. Here we show that lucicebtide exposure reprograms human immunosuppressive M2-like macrophages to a pro-inflammatory M1-like phenotype, restores cytotoxic T cell activation in immunosuppressed co-culture assays *in vitro*, and further increases T-cell activity in M1-like/T cell co-cultures. In immunocompetent, macrophage-rich triple-negative breast and colorectal cancer models, lucicebtide induces repolarization of tumor-associated macrophages (TAMs) to a pro-inflammatory M1-like phenotype and suppresses tumor growth. Lucicebtide synergizes with anti-PD-1 therapy and overcomes resistance to checkpoint inhibition in anti-PD-1-refractory tumors, but *in vivo* responses are impaired by systemic macrophage depletion, indicating that macrophage reprogramming is integral to lucicebtide activity. These results identify lucicebtide as a novel immunomodulator that reprograms immunosuppressive macrophage populations to enhance anti-tumor activity and suggests its utility for combination strategies in cancers with poor response to ICIs.

## Introduction

Immune checkpoint inhibitors (ICIs) have achieved clinical success with durable responses, but their efficacies are limited to a subset of patients (1). ICIs inhibit negative regulators of T cell responses, such as PD-1 or CTLA-4 (2), to restore cytotoxic T cell (CTL) activity against tumor cells and have significantly changed the clinical outcomes of aggressive cancer types such as melanoma. In contrast, tumors including glioblastoma (GBM) (3), breast cancer (BC) (4), ovarian cancer (5), pancreatic ductal adenocarcinoma (6), and colorectal cancer (7) are characterized by immunosuppressive TMEs that cause immune exclusion and impair responses to ICIs (2, 8). As immune exclusion is a primary obstacle to the success of ICI therapy, targeting the immunosuppressive TME has been identified as a promising combination strategy for cancers with poor response to ICI therapy alone.

Tumor-associated macrophages (TAMs) are primary contributors to the immunosuppressive TME (9). In physiological conditions, macrophages serve biological functions from promoting immune responses via antigen presentation and chemokine/cytokine secretion, to suppressing immune responses in the context of wound healing. In tumor biology, TAMs secrete cytokines and chemokines that suppress antitumor immunity and promote tumor progression. An M1/M2 paradigm describing a broad spectrum of macrophage transcriptional programs, ranging from the immune-promoting “M1-like” to the immunosuppressive “M2-like” has been proposed (10). Consistent with promoting tumor growth, metastasis, and therapeutic resistance, TAMs are mostly associated with the M2-like program, although differences among tumor types have been reported (11). Therapies aimed at reprogramming TAMs toward an M1-like program are therefore predicted to sensitize intractable tumors to immune therapies (12).

C/EBPβ is a transcription factor with a direct role in regulating tumor cell growth, proliferation, and metabolic switching (13, 14). In addition, C/EBPβ promotes a transcriptional program in monocytes linked to the M2-like phenotype (15, 16). Specifically, C/EBPβ was demonstrated to regulate expression of M2 target genes *Msr1*, *Il10* and *Il13ra* (17) in an *in vitro* model of murine macrophage differentiation. These data suggest that therapeutic suppression of C/EBPβ activity may repolarize macrophages away from the M2 immunosuppressive phenotype and yield clinical benefit in tumors resistant to immunotherapy due to an immunosuppressive TME. Lucicebtide (previously referred to as ST101) is a peptide antagonist of C/EBPβ (18) that is currently Phase 3-ready following completion of a Phase 2 study in patients with GBM (Clinicaltrials.gov ID: NCT04478279). Lucicebtide disrupts C/EBPβ dimerization, thereby enhancing its proteasomal degradation, resulting in significant attenuation of C/EBPβ target gene expression (18). Lucicebtide exposure results in direct anti-cancer activity both *in vitro* and *in vivo* in several preclinical models (18), and has shown promising activity in the clinic.

Here we investigated the impact of pharmacological antagonism of C/EBPβ with lucicebtide on human peripheral blood mononuclear cell (hPBMC)-derived macrophage cultures *in vitro* and on TAMs in syngeneic mouse tumor models *in vivo*. We demonstrate that lucicebtide promotes an M1-like program in hPBMCs cultured in conditions that should drive M2-like polarization and that short-term lucicebtide exposure to already activated and established M2-like macrophages is sufficient to repolarize toward an M1-like phenotype. These data support macrophage polarization as a plastic event, which, at least in part, is controlled by C/EBPβ. In co-culture of M2-like macrophages with CD8+ T cells, lucicebtide restores CTL activity as determined by increasing the fraction of Interferon-Gamma (IFN-γ) expressing T cells. Lucicebtide similarly impacts macrophage polarization *in vivo*, increasing the M1/M2 ratio of murine TAMs in an orthotopic triple negative breast cancer (TNBC) syngeneic model. To demonstrate the impact of lucicebtide-mediated macrophage polarization *in vivo*, combination of lucicebtide with anti-PD1 checkpoint inhibition resulted in enhanced anti-tumor activity compared to either single agent in an anti-PD-1 refractory TNBC model. Taken together, these data identify lucicebtide as a novel immune modulator of the TME with the potential to overcome resistance to ICIs or enhance their anti-tumor activity.

## Material and methods

### Peptide synthesis

Lucicebtide (*H_2_N*-vaeareelerlearlgqargelkkwkmrrnqfwlklqr-*OH*) was synthesized by Fmoc solid-phase peptide synthesis (SPPS) and the mass and sequence were confirmed by mass spectrometry. Lucicebtide solution was prepared from lyophilized powder in sterile milli-Q H_2_O containing 270 mM trehalose to a stock concentration of 2 mM.

### M1/M2 macrophage derivation and culture

Healthy human donor PBMCs were isolated from Human Leukomax leukopaks (BioIVT) by Ficoll Paque Plus density gradient according to the manufacturer protocol (Cytiva # 17-1440-02). Monocyte concentrations were estimated by flow cytometry using an FSC/SSC Monocyte gate. Cells were seeded at 1 million/cm^2^ in Monocyte Attachment Media (PromoCell # C-28051) and incubated 2 hrs at 37°C. Cells were retrieved, washed with Attachment media and replenished with 5 mL M1 (Promocell #C-28055) or M2 Generation Medium (#C-28056). For the long-term protocol, drug or controls were added on day one. Cultures were replenished with three mL M1 or M2 media on day six, and on day seven, M1 cells were activated by addition of 10 ng/mL LPS (Sigma #L2360) and 50 ng/mL IFN-γ (R&D Systems #285-IF-100), or M2 cells were activated by 20 ng/mL IL-4 (R&D Systems #204-IL-010). On day nine, the floating fraction of each culture was recovered, spun down, and added to the original flask in M1 or M2 media with the cytokine supplementation described above and drug treatment as indicated by the protocol. For the long-term protocol, cells were analyzed on day 10. For the short-term protocol, Lucicebtide or controls were added on day 10 and immunophenotype analyzed on day 13.

### Macrophage and T cell co-cultures

CD4+ or CD8+ T cells were sorted from hPBMCs by negative magnetic selection (Dynabeads Untouched Human CD8 T Cells Kit ThermoFisher #11348D; Dynabeads Untouched Human CD4 T Cells Kit ThermoFisher #11346D). Sorted cells were maintained at 5E5 cells/mL in RPMI media supplemented with 10% FBS, 55 µM β-mercaptoethanol, 10 ng/mL IL-2 (R&D Systems #202-IL-010) and 10 μL/mL Dynabeads Human T-Activator CD3/CD28 (ThermoFisher # 11161D). M1 and M2 cultures were established and activated as described above. For the co-culture assay, M1 and M2 media were removed and T cells were plated at 2.5E5 cells/mL. T cells were recovered three days later and stained for intracellular IFN-γ as described below.

### Quantitative RT-PCR

Cells treated with lucicebtide for the indicated times were resuspended in RNAlater (Thermo Fisher Scientific, USA) and total RNA was extracted using Qiagen RNeasy Protect Mini Kit (Qiagen Inc. USA) according to manufacturer’s instructions and treated with Qiagen RNase-free DNase to remove genomic DNA. RNA quality and quantity were measured by Nanodrop, agarose gel electrophoresis and Agilent 2100 Bioanalyzer. For qPCR, cDNA was synthesized from total RNA using SuperScript IV VILO Master Mix with ezDNAse enzyme (Thermo Fisher Scientific, USA) per manufacturer’s protocol. qPCR reactions were run on a QuantStudio 6 Flex real-time thermal cycler in quadruplicate using 10 ng cDNA, gene-specific primers (0.15 µM each) and Fast SYBR™ Green Master Mix (Thermo Fisher Scientific, USA). Primers sequences are listed in **Supplementary Table S1**. Control reactions without reverse transcriptase were performed. Data was analyzed assuming 100% PCR efficiency. Log2 normalized expression (2^ΔΔCt) and standard error of mean were used to determine fold change of expression in relation to β-actin between treated and untreated samples (19). Statistical significance between groups was determined using Student’s T-test.

### Flow cytometry

The following fluorochrome conjugated antibodies were purchased from Biolegend: APC-Human-CD68 (#333810), BV421-Human-CD163 (#333612), FITC-Human-CD80 (#305206), PE-Human-IFNG (#506507), BV421-Mouse-CD206 (#141717), FITC-Mouse-CD80 (#104706), APC-Mouse-CD11b (#101212), BV650 CD45 (#103151) and FITC-mouse-I-A/I-E (#107606). For hPMBCs and T cell staining, single cell suspensions were recovered from cultures (for macrophage cultures both the suspension and adherent fractions were collected by scraping), washed in FACS Buffer (0.1% FBS in PBS) and resuspended in 50 μL FACS Buffer supplemented with 5 μL Human TruStain FcX (BioLegend #422302) at a concentration of 1E6 cells/mL. Five μL of the indicated Ab were added and cells were incubated 20 minutes at 4C in the dark. For macrophage staining, antibodies were supplemented by 5 µl of True-Stain Monocyte Blocker (Biolegend #426102). Cells were then washed twice in 200 μL FACS buffer, resuspended in 400 μL and acquired on a MACSQuant (Miltenyi) or FACS Celesta (BD) flow cytometer. Mouse tumors were collected by resection, reduced to single cell suspension by use of a 70 μM mesh and resuspended in FACS Buffer supplemented with 1 mM EGTA 1mM and 0.02 mg/mL DNAse (Sigma) at a concentration of 1E6 cells/mL. Fifty μl of cell suspension were incubated with 5 μl TruStain FcX™ PLUS (BioLegend #156604) for 5 minutes at 4C. For surface antigens, antibody staining, wash and sample acquisition were performed as above. For IFN-γ intracellular staining, cells were permeabilized following surface staining by resuspension in 100 µL Cyto-Fast Fix Perm Solution (Biolegend #426803) for 20 minutes at RT. Cell were then washed in 1X Cyto-Fast Perm Wash solution and stained in 100 µL Cyto-Fast Perm Wash Solution supplemented with 2.5 µL PE-Anti-IFNγ. After incubating 20 minutes at room temperature in the dark, cells were washed twice by adding 100 µl of Perm Wash solution and once in FACS Buffer and acquired on a MACSQuant Flow Cytometer.

### Gene expression analysis and TCGA-BC classification

The CEBPB_01 (CEBPB_01 (gsea-msigdb.org)) and CEBPB_02 (CEBPB_02 (gsea-msigdb.org)) gene sets were merged to compile a CEBPB-bound list of 473 unique genes that present the motif RNRTKDNGMAAKNN (CEBP_01) or NKNTTGCNYAAYNN (CEBP_02) in the regions spanning +/- 2KB centered on their transcription starting sites (**Supplementary Table S2**). RNAseq profiles of HR-negative TCGA BC samples were scored for the CEBPB-bound signature. HR status was derived from Ciriello et al., 2015. Samples were then stratified into the top quartile (Top25; n=124) or bottom three quartiles (Lower75; n=369). Cases with no survival data were removed. Survival difference was assessed by log-rank (Mantel-Cox) test.

### Immune infiltration and CEBPB signature scoring

RNAseq profiles for ovarian cancer and GBM were retrieved from TCGA and scored for CEBPB transcript level in Lower75 and Top25 sets as described above. Samples with available Immune Infiltrate Macrophage, M1 and M2 XCell scores (ovarian cancer, n=306; GBM, n=165) from Timer 2.0 app (cistrome.timer.org) were classified into High or Low sets according to the median. Associations between Top25 and Lower75 sets and High/Low signatures were calculated by Fisher T-test.

### RNAseq profiling and differential expression analysis

M2 cultures were derived and activated as described above in the presence of lucicebtide 10 μM or left untreated as control. On day 10, samples were collected by recovering both the floating and adherent fraction by scraping. Cell were sorted for viability and CD68 positivity with gates including both CD68 low and high populations. Total RNA was extracted by Qiagen RNeasy columns with the addition of DNase treatment to remove genomic DNA. RNA libraries were processed as described previously (18). For RNAseq analysis, genes with transcript level less than 2 TPM in 4 or more samples, mitochondrial or immunoglobulin transcripts were removed. Unsupervised Clustering (UC) and differential expression analysis were performed with Matcalc software (20). For UC analysis, transcript levels were log2-transformed and genes with standard deviation greater than 1.5 were used. Differentially expressed genes (DEG) were defined as greater than 1.5 fold change and Student t-test p-value less than 0.05 after Benjamini-Hochberg correction. Data are available as GEO dataset GSE288861. GSEA (21) was performed for gene sets from MySigDB C2 collection using signal-to-noise statistics and gene-set permutations.

### Mouse tumor models

4T1 cells (4x10^6^) were mixed 1:1 with Matrigel and injected in the fourth mammary pad of syngeneic 6-8 wks old Balb/C female mice. Tumors were tracked by tri-weekly caliper measurements. One-week after injection, mice with tumors greater than 60 mm^3^ were randomly assigned to treatment or control cohorts. Lucicebtide was administered subcutaneously at the indicated dosages three times weekly. CT-26 cells (1x10^5^) were mixed 1:1 with Matrigel and injected in the right flank of Balb/C female mice. Mice were staged upon reaching tumor volumes greater than 200 mm^3^ and were assigned to control or treatment cohorts. For the combination studies, anti-mouse-PD-1 (BioXCell #BE0146, clone 29F.1A12) was administered weekly at 12.5 mg/kg while lucicebtide was administered subcutaneously at 25mg/kg or 10 mg/kg three times weekly. All aspects of animal care were in accordance with the Guide for Care and Use of Laboratory Animals and all experiments were approved by the Institutional IACUC at New York Medical College (NYMC).

## Results

### Lucicebtide inhibits macrophage polarization to the M2 program in hPBMC–derived macrophage cultures

**Figure 1:**
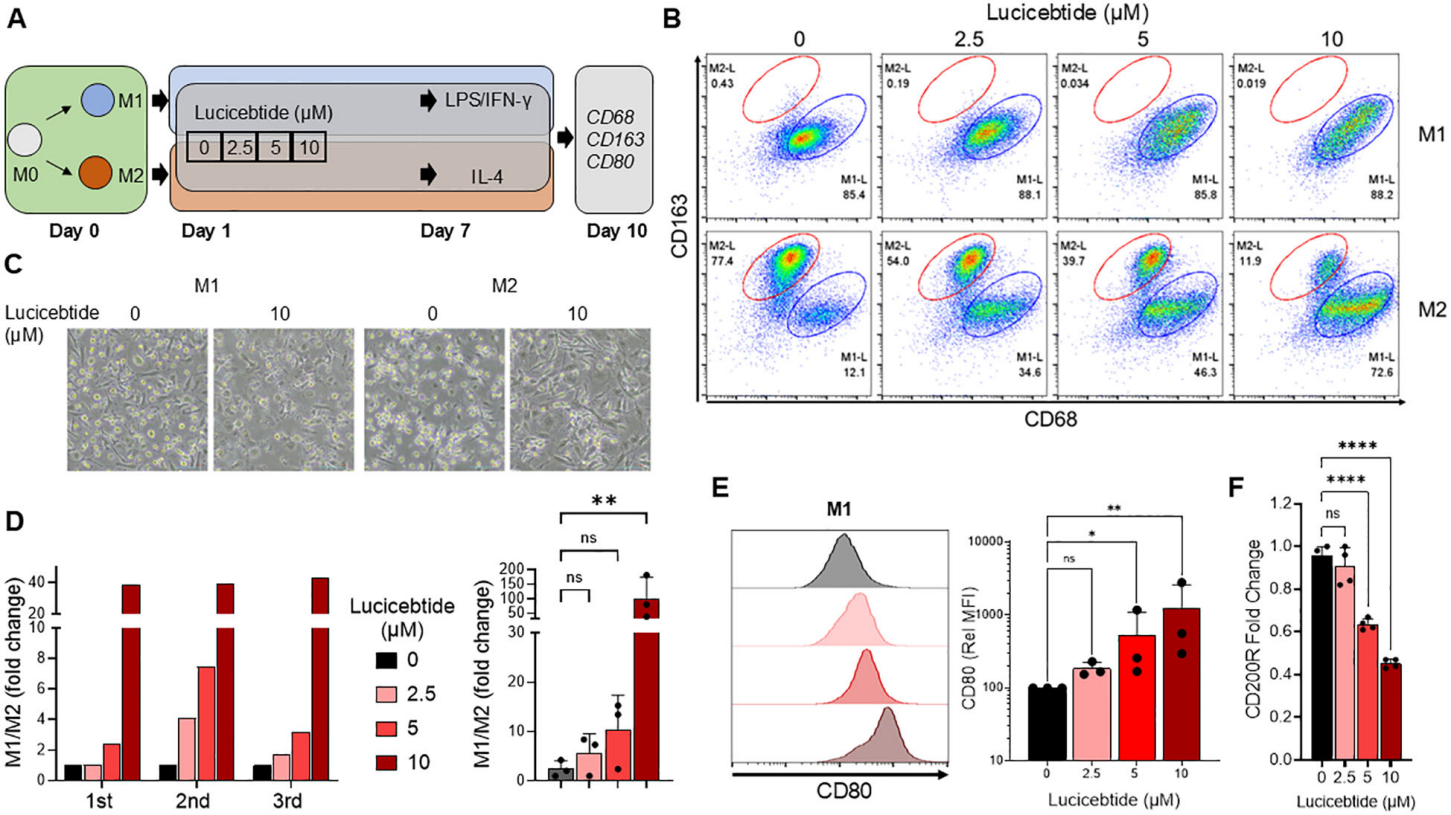
Lucicebtide shifts the M2 program to M1 in Human PBMCs–derived macrophage cultures. **(A)** Experimental outline describing derivation of M1 and M2 macrophages from hPBMCs. **(B)** Flow Cytometry plots of M1 (top) and M2 (bottom) populations at the indicated lucicebtide concentration on Day 10. M2 populations (red) are gated as CD68^low^CD163^high^, M1 populations (blue) are CD68^high^CD163^low^. **(C)** Bright Field images of M1 and M2 culture untreated or treated with 10 μm lucicebtide. Scale bar, 50 μm. **(D)** Left, hPBMCs-derived M1/M2 population ratios for three donors at the indicated lucicebtide concentrations in Day 10 M2 cultures. Data are normalized to the untreated. Right, averages of the three donor derived cultures at the indicate concentrations. Error bars represent SDs. Statistics, 1-way ANOVA, Student t-test (n=3/group; ns, not significant, **p<0.01) **(E)** Left, histogram plots for CD80 in Day 10 M1 cultures at the indicated lucicebtide concentrations (left). Right, relative CD80 MFI at the indicated lucicebtide concentrations. MFI was normalized to the untreated for each donor. Error bars represent SDs. Statistics represent 1-way ANOVA (n=3/group; *p<0.05; **p<0.01; ns, not significant). **(F)** Expression levels of CD200R normalized to ACTB at the indicated lucicebtide concentrations for Day 10 M2 cultures. Error bars represent SEMs. Statistics represent Student T-tests (n=4 replicates; ****p<0.001; ns, not significant 1-way ANOVA).

Lucicebtide antagonizes C/EBPβ dimerization, promoting its proteasomal degradation and inhibiting C/EBPβ transcriptional activity (18). To investigate the impact of C/EBPβ antagonism on macrophage polarization, macrophage cultures from the peripheral blood of three healthy human donors were established, and differentiation and activation toward the M1-like (referred to as ‘M1’) or M2-like (referred to as ‘M2’) phenotype was induced in the presence of increasing concentrations of lucicebtide (**Figure 1A**). After a seven-day incubation in M1 or M2 induction media, macrophages were activated with LPS + IFN-γ (M1) or IL-4 (M2) and subsequently analyzed for surface markers by flow cytometry on day 10. M1 cells were gated as CD68^high^CD163^low^ and M2 as CD68^low^CD163^high^ (**Figure 1B**). For all three donors, lucicebtide increased the M1:M2 ratio in macrophage cultures stimulated to the M2 phenotype in a dose-dependent manner, with a relative ratio increase of 40-fold compared to control at the highest lucicebtide concentration (**Figures 1C, D**). Remarkably, despite CD80^high^ expression, lucicebtide exposure led to a dose-dependent increase of CD80 median fluorescence intensity (MFI) in macrophage stimulated to the M1 phenotype, suggesting that lucicebtide further augments the M1 program in M1 cells (**Figure 1E**). Conversely, in the M2 population, the M2 marker CD200R was down-regulated in a dose-dependent manner compared to untreated M2 cultures (**Figure 1F**). Importantly, no substantial reduction of total viable cell numbers was observed in M1 or M2 cultures following lucicebtide exposure (**Supplementary Figure S1**). These data support the ability of lucicebtide to promote the M1 program upon continuous exposure during M2 commitment and activation.

The impact of lucicebtide on macrophage plasticity was investigated in the ex vivo system. In one set of experiments, macrophage cultures were induced to M2 phenotype as described, followed by addition of lucicebtide on day 10 and evaluation for M1/M2 markers on day 13. In a second set of experiments, M2 macrophages were exposed to lucicebtide during activation toward the M2 phenotype as in initial experiments, followed by washing away lucicebtide on Day 10. Cells were then resuspended in control media or media containing 10 µM lucicebtide, and evaluated on day 13 (**Supplementary Figure S2A**). Control M2 cells were left untreated or exposed to lucicebtide for the entire experiment. As previously observed, continuous lucicebtide exposure induced an almost complete shift toward the M1 identity, with a 250-fold shift in the M1/M2 ratio compared to untreated M2 cultures. Lucicebtide addition on day 10 induced a 34-fold shift in the M1/M2 ratio compared to untreated M2 cultures (**Supplementary Figures S2B, C**), indicating that C/EBPβ activity is critical for maintaining the M2 program and that lucicebtide can convert established M2 cultures to the M1 identity. Withdrawal of lucicebtide from cultures had minimal impact, with a 21-fold M1/M2 ratio increase observed compared to control on day 13. Overall these observations indicate that macrophage polarization is a plastic event, that C/EBPβ activity is necessary for establishing and maintaining the M2 program, and antagonism of C/EBPβ with lucicebtide can both instruct the M1 program in M2 conditions and convert immune-suppressive M2 macrophage to the immune-active M1 program.

### Lucicebtide suppresses C/EBPβ target genes and M2 programs

To investigate lucicebtide impact on C/EBPβ target genes and the M2 program, M2 cultures were exposed to 10μM lucicebtide or control as described above, and cultures were collected on day 10 for RNAseq analysis. Unsupervised clustering (UC) identified a 45 gene signature that distinguished control from lucicebtide-treated cells (**Supplementary Figure S3A**), notably including several M2 markers or factors involved in TAM biology, including ALDH1A2 (22), CD93 (23), FOLR2 (24), MARCO (25) and the chemokines CCL17, CCL24, CCL13 and CCL17 (26) (**Supplementary Table S3**). Differential expression analysis exposure revealed a total of 414 DEGs genes, including 248 downregulated and 166 upregulated following lucicebtide exposure (**Supplementary Figure S3B**, **Supplementary Table S4**). Consistent with lucicebtide activity on C/EBPβ target genes in cancer cells (18), ID2, BIRC3, CyclinA2 and CDK1 were significantly downregulated in lucicebtide-treated M2-cells (**Supplementary Figure S3C**). GSEA analysis (21) of the RNAseq dataset identified significant downregulation of cytokine/chemokine and NF-kB signaling pathways and an increase in genes implicated in activation of steroid synthesis (**Supplementary Figure S3** **;** **Supplementary Table S5**).

### Lucicebtide promotes T cell activation in immunosuppressive M2 co-cultures

**Figure 2:**
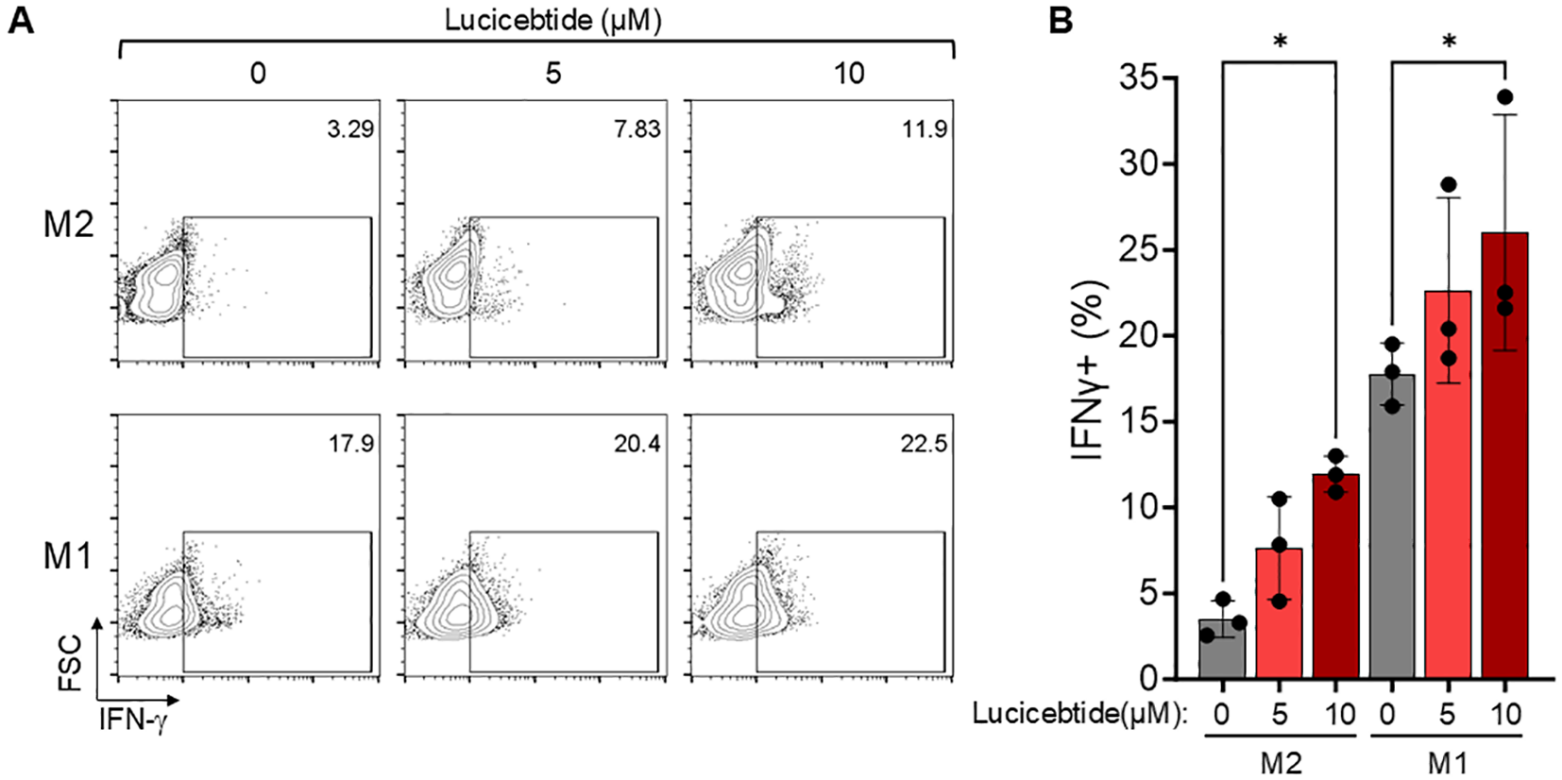
Lucicebtide-mediated M2 to M1 conversion results in enhanced T-cell activation. **(A)** Representative flow-cytometry plots for intracellular IFN-γ staining in the indicated conditions (top, M2; bottom, M1; from left to right 0,5 and 10 µM lucicebtide). Cell frequency is shown in the top right corner. **(B)** IFN-γ frequency for the indicated conditions. Error bars represent standard deviations. Statistics represent 1-way ANOVA (*p<0.05; n=3/group).

Suppression of T cell proliferation and activation has been shown following co-culture with M2 macrophages *in vitro* (27). To investigate whether lucicebtide-mediated M2-to-M1 conversion will enhance CD8+ T cell activation *in vitro*, M2 and M1 cultures from normal hPBMCs were derived and activated, as described above, and co-incubated with CD8+ T cells sorted and expanded from the same donors. M2 or M1 cells were exposed to 5 or 10 µM lucicebtide or control for 72 hrs, after which time media was removed and cells were co-incubated with CD8+ T cells. After three days of co-culture, T cells were recovered and intracellular IFN-γ measured by flow cytometry. As expected, the frequency of IFN-γ^+^ T cells in co-culture with immunosuppressive M2 macrophages was five-fold less than in co-culture with M1 macrophages (3.5% *vs* 17.8%, **Figures 2A, B**). Lucicebtide induced a dose-dependent increase in the frequency of IFN-γ^+^ fraction cells in M2 co-cultures (5 and 10 µM lucicebtide induced a 2.2- and 3.4-fold increase in IFN-γ^+^ cells, respectively; p<0.05,1-way ANOVA) or M1 co-cultures (5 and 10 µM lucicebtide induced a 27% and 46% increase of IFN-γ^+^ cells, respectively; p<0.05,1-way ANOVA; **Figures 2A, B**). Notably, 10 µM lucicebtide restored the frequency of IFN-γ^+^ cells in M2 co-cultures to 67% of what was observed in M1 co-cultures. These results suggest that lucicebtide exposure is sufficient to restore T-cell activity in the presence of M2 macrophages and can further enhance T-cell response in immune-active conditions.

### The C/EBPβ gene signature correlates with poor prognosis in breast cancer

**Figure 3:**
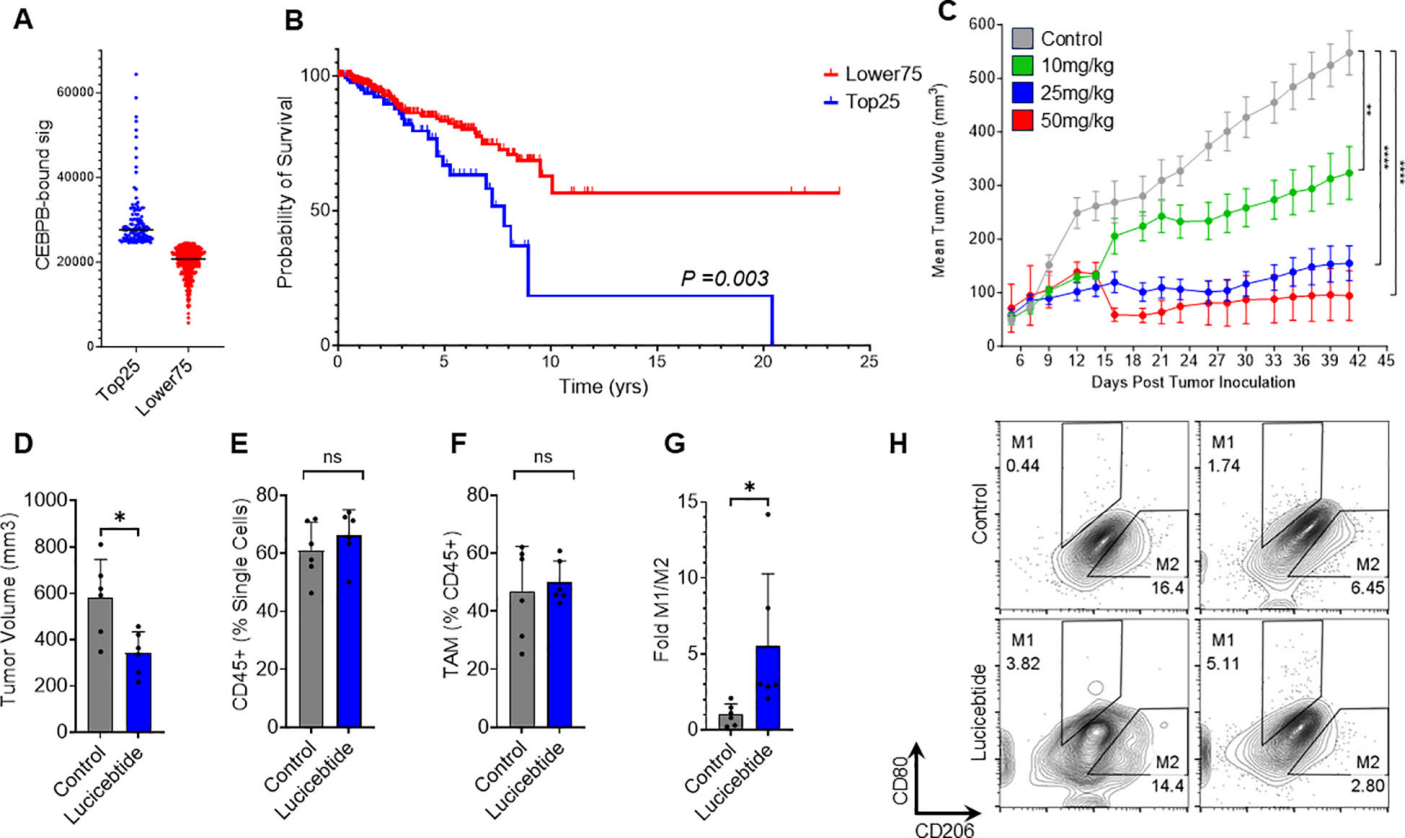
Lucicebtide antagonism of C/EBPβ promotes M2 to M1 repolarization *in vivo*. **(A)** Distribution of TCGA HR-Negative Breast Cancer Samples according to the CEBPB-bound signature (Top25, blue, n=124; Lower75, red, n=369). Horizontal lines indicate score averages. **(B)** Survival curves for the Lower75 and Top25 sets. Statistics indicate Log-rank test. **(C)** Tumor volumes of mice transplanted with 4T1 cells and treated with vehicle (gray) or lucicebtide at the indicated dosing (green, 10 mg/kg; blue 25 mg/kg; red, 50mg/kg). Statistics indicate 1-way ANOVA (****p<0.0001; **p<0.01; n=6/group). **(D)** Tumor volumes for mice treated with Vehicle (gray) or 25 mg/kg lucicebtide (blue) and resected 16 days after treatment inceptions. Error bars represent standard deviation (*p<0.05, Student t-test, n=7/group). **(E, F)** Fraction of CD45^+^ cells out of total single cells **(E)** and Fraction of TAMs out of CD45^+^ cells **(F)** in tumors treated with vehicle or lucicebtide (ns, not significant, Student t-test, n=6/group). **(G)** Fold M1/M2 ratios of TAMs for control and lucicebtide-treated tumors. Data are normalized to the control average (*p<0.05, Student t-test, n=6/group). **(H)** Flow cytometry plots of M1 (CD80^high^CD206^low^) and M2 (CD80^low^CD206^high^) gating of TAMs for two control and lucicebtide-treated tumors. The samples with highest and lowest M1/M2 ratios for control and lucicebtide-treated cohorts are shown.

Elevated TAM levels are linked to poor prognostic outcomes in several cancer types (28). In BC, high TAMs density is associated with inferior prognosis (29), leading to the pan-macrophage marker *CD68* included among the 16 genes used in the Oncotype DX Breast Recurrence Score diagnostic (30). To investigate the relevance of C/EBPβ-driven immunosuppressive programs in human cancers, we assessed whether C/EBPβ signatures correlate with patient outcomes in BC. To do that, we defined a *C/EBPβ* gene set by merging two existing MySigDB sets including genes with *C/EBPβ* binding sites +/- 2KB with respect of their transcription starting site (21) (**Supplementary Table S2**). This 473-gene set was used to score profiles from TCGA HR-negative BC samples (31) from the Cancer Gene Atlas (TCGA). Kaplan–Meier analysis of the top quartile (Top25) compared to the lower three quartiles (Lower75) was performed. The Top25 showed a markedly inferior prognosis (median survival = 7.8 yrs) compared to the Lower75 (undefined median survival; p=0.003, Log-Rank test), indicating that expression of the *C/EBPβ* signature inversely correlates with survival in HR-negative BC (**Figures 3A, B**). Similarly, the *C/EBPβ* signature inversely correlates with survival in HR-positive BC (**Supplementary Figure S4A, B**). These data support a prognostic value for C/EBPβ-dependent programs in BC and identifies the potential for lucicebtide reprogramming of the immunosuppressive TME in TAM-rich cancers.

### Lucicebtide promotes M2-to-M1 repolarization *in vivo*

We have previously demonstrated lucicebtide anti-tumor activity in xenograft models of C/EBPβ-driven cancer cells in mice lacking an active immune system (18). To investigate the impact of lucicebtide on TAM populations and immune-mediated anti-tumor responses, we utilized the 4T1 TNBC orthotopic model in BALB/C syngeneic hosts, which enables the study of tumor responses in the context of a competent immune system (32). Consistent with clinical BC, this model is characterized by high immunosuppressive TAM content (33). Initial experiments assessed the anti-tumor activity of monotherapy lucicebtide following administration of 10, 25 or 50 mg/kg three times weekly for the duration of the study, resulting in 45.6%, 73.8% and 95.4% tumor growth inhibition (TGI), respectively (1-way ANOVA between tumor volumes at the indicate time points, **p<0.01; ****p<0.0001; n=6 mice/group, **Figure 3C**). No significant impact of lucicebtide on mouse body weight was observed (**Supplementary Figure S5**). To assess the impact of lucicebtide on the TAM population in this model, we resected control and lucicebtide-treated (25 mg/kg, 3 times weekly) tumors after 16 days of treatment and analyzed TAM content and polarization by flow cytometry. On day 16, lucicebtide induced a 41% reduction in tumor volume compared to control (*p<0.05, Student t-test n=6/group; **Figure 3D**). While the proportion of CD45+ cells (as fraction of single cells) and total TAM (as fraction of CD45+) were not significantly impacted, lucicebtide induced a 5.5-fold increase in M1/M2 ratio compared to control (*p<0.05, Student t-test, n=6/group; **Figures 3E-H**). This data indicates that lucicebtide promotes repolarization of immunosuppressive TAMs toward immune-active M1 program and an enrichment in M1 macrophages *in vivo*.

### Macrophage infiltration correlates with high *C/EBPβ* transcript levels

To investigate the relationship between *C/EBPβ* and macrophage infiltration in additional cancer types, RNAseq profiles of ovarian cancers and GBMs from the TCGA repository were scored for *C/EBPβ* transcript level and stratified into the top 25% and lower 75%. The presence of macrophage, M1 and M2 infiltrates were assessed using Xcell estimates by Timer 2.0 (34, 35) and tumors were classified as high or low macrophage infiltration based on median values. The association between C/EBPβ transcript expression and macrophage infiltration was assessed by Fisher T-test (**Supplementary Table S6**, **Supplementary Figure S6**). Ovarian tumors that were within the Top25 for C/EBPβ transcript were significantly enriched with all macrophage signatures. Top25 GBMs were enriched for total macrophage and M1 signature (**Supplementary Figures S6B, C**). These results suggest that C/EBPβ expression is positively correlated with macrophage infiltration in human tumors. Better characterized M1 and M2 signatures may be needed to appreciate the specific impact of C/EBPβ expression on these populations.

### Lucicebtide enhances anti-tumor activity of anti-PD-1 therapy

**Figure 4:**
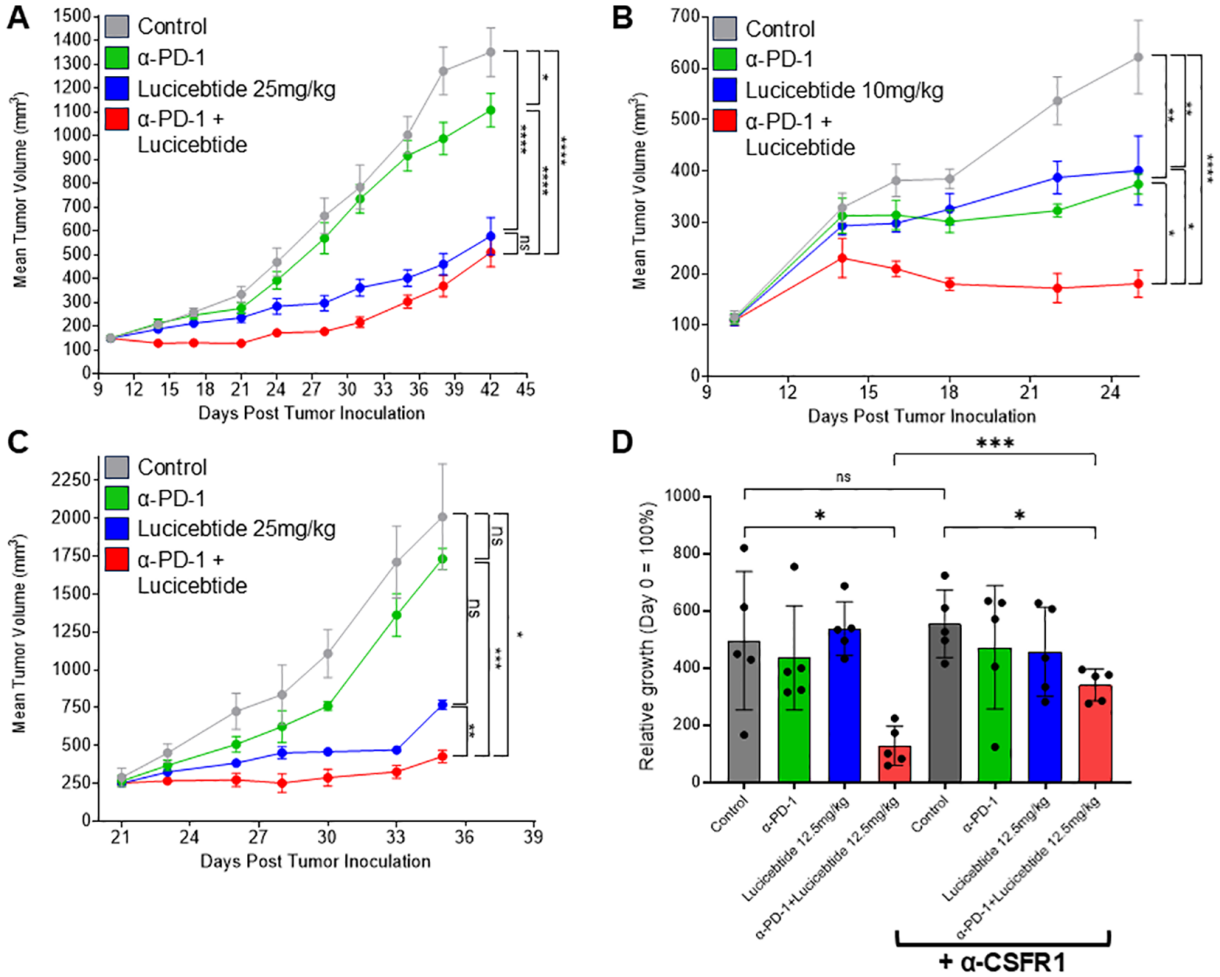
Lucicebtide enhances anti-tumor activity of anti-PD-1. **(A)** Tumor volumes of mice transplanted with 4T1 cells and treated with vehicle (gray) or with the indicated treatment (lucicebtide, 25 mg/kg, blue; anti-PD-1, green; combination, red). Statistics indicate 1-way ANOVA (****,p<0.001;*,p<0.05; ns, not significant; n=10/group) for volumes at Day 42. **(B)** Tumor volumes of mice transplanted with 4T1 cells and treated with vehicle (gray) or with the indicated treatment (subpharmacologic lucicebtide, 10mg/kg, blue; anti-PD-1, green; combination, red). Statistics indicate 1-way ANOVA (****,p<0.001; **,p<0.01; *,p<0.05; n=7/group) for volumes at Day 25. **(C)** Tumor volumes of mice transplanted with anti-PD-1 resistant 4T1R cells and treated with vehicle (gray) or with the indicated treatment (lucicebtide, 25 mg/kg, blue; anti-PD-1, green; combination, red). Statistics indicate 1-way ANOVA (***,p<0.001; **,p<0.01; *,p<0.05; ns, not significant; n=3/group) for volumes at Day 35. **(D)** Relative growth rates for 4T1R tumors with the indicated treatments (n=5/group): vehicle, dark gray; anti-PD-1, green; 12.5 mg/kg lucicebtide, blue; anti-PD-1 and lucicebtide combination, red; α-CSFR1 bracket indicates supplementation with anti-CSFR1 antibody in addition to the indicated treatment. Statistics indicate 1-way ANOVA (***,p<0.001; *,p<0.05; ns, not significant) for relative growth rate at Day 19.

To investigate the impact of lucicebtide repolarization of macrophages on anti-tumor responses, studies were performed in combination with ICI therapy in the 4T1 TNBC model. Mice bearing orthotopic mammary 4T1 tumors were treated with vehicle, anti-mouse-PD-1 (12.5 mg/kg, once weekly), lucicebtide (25 mg/kg, 3x/week) or administered both drugs in combination (**Figure 4A**). On day 42, single-agent lucicebtide induced a 64.2% TGI (p<0.0001 *vs*. control) and anti-PD-1 induced a 20.3% TGI (p=0.0365 *vs*. control), consistent with literature indicating poor anti-PD-1 responses in the 4T1 model (33). The combination cohort showed a 70.0% TGI, significantly greater than the anti-PD-1 response (p<0.0001 *vs*. anti-PD-1), however only modestly improved from lucicebtide alone (p=0.5466 *vs*. lucicebtide). To assess the impact of the immune-modulatory activity of lucicebtide without engaging its direct anti-tumor activity, mice bearing orthotopic 4T1 tumors were treated with subpharmacologic lucicebtide (10 mg/kg, 3x/week), anti-mouse-PD-1 (12.5 mg/kg, once weekly) or both drugs in combination (**Figure 4B**). In this setting, partial responses were observed on day 25 in the single agent arms (anti-PD-1: 48.1% TGI, p=0.0025 *vs*. control; lucicebtide: 42.5% TGI, p=0.006 *vs*. control) while the combination cohort displayed a greater suppression of tumor growth (85.8% TGI, p<0.0001 *vs*. control,1-way ANOVA). In this subpharmacologic lucicebtide setting, the combination treatment displayed significantly enhanced activity compared to either single agent alone (p=0.0142 *vs*. anti-PD-1; p=0.0061 *vs*. lucicebtide; 1-way ANOVA, **Figure 4B**). In the syngeneic CT26 CRC model in immuno-competent Balb/c mice, lucicebtide (25 or 50 mg/kg) resulted in 49.3% and 66.7% TGI (1-way ANOVA of *p<0.05 and **p<0.01; n=5 mice/group; **Supplementary Figure S7A**). Similar to the 4T1 model, subpharmacologic lucicebtide administered in combination with anti-PD-1 in the CT-26 model resulted in enhanced combination efficacy (**Supplementary Figure S7B**). Overall, these results support the potential clinical relevance of lucicebtide in combination with anti-PD-1 therapy.

### Lucicebtide overcomes resistance to anti-PD-1 therapy

We next sought to define lucicebtide activity in tumors that do not respond to anti-PD-1 therapy. Since partial responses are observed in parental 4T1 tumors (**Figure 4B**), we derived a refractory line by secondary-transplanting a 4T1 tumor that did not respond to anti-PD-1 treatment (4T1R). Consistent with these cells displaying resistance to anti-PD1, 4T1R tumors do not display a statistically significant response to anti-PD-1 treatment (13.8% TGI, p=n.s. *vs*. control, **Figure 4C**). On the contrary, 25 mg/kg lucicebtide potently suppressed 4T1R tumors (61.7% TGI, p=0.07 *vs*. control). The combination of anti-PD-1 and lucicebtide resulted in a 78.7% TGI compared to vehicle-only control (p<0.05 *vs* control). Importantly, the combination significantly suppressed tumor growth when compared to single-agent responses (*vs*. lucicebtide alone; 44.3% TGI, p<0.01; *vs*. anti-PD-1 alone: 75.3% TGI, p<0.001, **Figure 4C**), indicating that combination with lucicebtide can overcome resistance to anti-PD-1 therapy.

### Macrophages are an integral component of Lucicebtide and anti-PD-1 combination activity

To demonstrate the relevance of lucicebtide’s impact on TAMs on the overall tumor response, a cohort of Balb/c mice bearing 4T1R orthotopic tumors were treated with anti-CSFR-1 antibody to deplete their macrophage population while a second cohort did not receive anti-CSFR-1 antibody and were utilized as controls. Mice were then administered vehicle control, subpharmacologic lucicebtide (12.5 mg/kg 3x/week), anti-PD-1 or anti-PD-1/lucicebtide combination as in previous experiments. Macrophage depletion was confirmed by flow cytometry as indicated by detection of reduced CD11b+ cells in each anti-CSFR-1 treatment cohort compared to the corresponding control (**Supplementary Figure S8**). Anti-CSFR1 alone did not substantially impact tumor growth in control animals (**Figure 4D**, p=0.643; see **Supplementary Figure S9** for tumor response curves) nor did anti-PD1 or subpharmacologic lucicebtide monotherapy. While combination lucicebtide and anti-PD-1 resulted in 74.2% TGI, administration of anti-CSFR1 treatment reduced the TGI in the combination group to 38.7% (**Figure 4D**, p<0.001), or a 52% reduction in response. These data support that the impact of lucicebtide on macrophages is a critical component of its anti-tumor responses.

## Discussion

Immunosuppressive TAMs are amongst the most prevalent innate immune cells within the tumor immune microenvironment and impact CTL activity both directly, via expression of checkpoint molecules and inhibitory cytokines that limit the activity of tumor infiltrating lymphocytes (TILs), or indirectly by recruiting Treg and inhibitory dendritic cells to the tumor. Due to these pro-tumorigenic properties, TAMs have become a target of immune oncology efforts (9). Interventions that inhibit TAM recruitment and/or deplete TAMs by targeting immune-modulatory receptors and ligand interactions, such as the colony-stimulating factor 1 (CSF1)/colony-stimulating factor 1 receptor (CSF1R) (36), CCL2 (monocyte chemoattractant protein‐1 (MCP‐1)/CCR2 axis (37), and the CXCL12/CXCR4 signaling axis (38), have shown encouraging, yet limited results. The discovery of genetic programs that control macrophage differentiation suggests that targeting macrophage polarization may offer a superior therapeutic approach (12), by enhancing proinflammatory M1 macrophage within the TME while selectively reprogramming immunosuppressive TAMs. Inhibition of PI3K-γ was introduced as a strategy to enable polarization of TAMs to a pro-inflammatory state and sensitize tumors to ICI therapy (39), however, PI3K-γ inhibitors have been shown to display opposite effects on macrophage polarization depending on the *in vivo* context (40), complicating their clinical utility. Further, dose-limiting toxicity and emerging resistance have limited the application of PI3K-γ inhibitors to date (41). Our data suggests that targeting signaling events downstream of PI3K-γ at the level of C/EBPβ enables specific inhibition of M2 macrophage polarization without toxicity.

Importantly, while a role for C/EBPβ in establishing an immunosuppressive M2 macrophage program has been shown (42), the role of C/EBPβ in maintenance of this phenotype is not understood. Our data identifying the plasticity of these programs and the ability of lucicebtide to antagonize C/EBPβ-driven gene expression demonstrates the potential of lucicebtide to reprogram macrophage populations toward an immune-active phenotype. Shifting the balance from immunosuppressive M2 macrophages to proinflammatory M1 macrophages represents a potential broad-application method to enhance the activity of existing immunotherapies such as ICIs. In our experiments, lucicebtide potently increased the M1/M2 macrophage ratio ex vivo, enabling CTL activation to levels observed by coincubation with proinflammatory macrophages. Surprisingly, lucicebtide further increased T cell activity in the presence of M1 cells, indicating that proinflammatory macrophages have the capacity to demonstrate enhanced immunostimulatory activity. Little is currently understood about increasing the proinflammatory potential of M1 macrophages in the clinic. We anticipate these findings to be especially relevant in tumors that are typically thought of as good candidates for ICI therapy due to high expression of PD-L1, yet yield poor responses to anti-PD-1 antibodies (43). Examples include tumors such as head and neck squamous cell carcinoma (HNSCC), metastatic melanoma and GBM (44). Specifically in the case of GBM, spatial transcriptomics identified CEBPB expression as a marker of M2-like macrophages, suggesting that antagonism of C/EBPβ would have a significant impact on the TME in this setting (45). Further supporting utility of lucicebtide for the treatment of GBM, our data suggests that lucicebtide regulates the expression of M2 markers in human microglia and increases the M1/M2 ratio in human GBM (unpublished results).

In summary, our study indicates that lucicebtide exposure promotes the loss of immune-suppressive M2 macrophages, with a concomitant increase in proinflammatory M1 macrophages. Macrophage polarization was shown to be a plastic event, as cells committed to the M2 program remained susceptible to lucicebtide-mediated repolarization. Macrophage polarization toward the M1 phenotype was demonstrated *in vivo* in an anti-PD-1-refractory syngeneic tumor model characterized by high TAM content, resulting in robust anti-tumor responses and enhanced anti-tumor responses in combination with anti-PD-1 checkpoint inhibition. Subsequent experiments in mice where macrophages were pharmacologically depleted confirmed the role of macrophage polarization in lucicebtide-mediated anti-tumor responses *in vivo*. Importantly, the impact of lucicebtide on macrophage polarization is likely to act in concert with its direct cytotoxicity in C/EBPβ-driven cancers and raises the potential that the target patient population for lucicebtide therapy may extend beyond only cancer types driven by C/EBPβ. These data support evaluation of lucicebtide-mediated antagonism of C/EBPβ in the clinical setting as an immune-modulatory agent to enhance the anti-tumor activity of immunotherapies such as checkpoint inhibitors.

## Data Availability

The original contributions presented in the study are included in the article/**Supplementary Material**. Further inquiries can be directed to the corresponding author. RNA-seq profiles of hPBMCs-derived M2-type macrophages treated with Lucicebtide are available as Geo Dataset series GSE288861.

## Supplementary material

Supplementary Figure 1 Lucicebtide does not reduce total cell numbers in M1 or M2 cultures. **(A-C)** Total viable cells reported for three independent hPBMC-derived M1 or M2 cultures exposed for 10 days to the indicated lucicebtide concentrations or left untreated. Averages of the relative viable cell number are shown for M1 **(D)** and M2 **(E)** lucicebtide-treated cultured from **(A-C)** ns, not significant by 1-way Anova compared to untreated.

Supplementary Figure 2 Lucicebtide repolarizes established M2 macrophages to the M1 identity. **(A)** Experimental outline of M2 culture establishment and activation from hPBMCs subject to the following treatments: untreated (gray box); untreated up to day 10 followed by 3-day exposure to lucicebtide 10 µm (blue); treated with lucicebtide 10 µm (violet); treated with lucicebtide 10 µM up to day 10 and switched to untreated media (cyan). All conditions were assessed by flow cytometry on day 13. **(B)** Flow Cytometry of Day 13 M2 cultures from the experimental condition described in A. M2 populations are manually gated as CD68^low^CD163^high^, M1 populations are CD68^high^CD163^low^. An M1 culture is shown for gating control. Statistics indicate percentages of parent population. **(C)** Log2 M1/M2 Ratio normalized to an untreated control cultures for the indicated condition. Statistics, 1-way Anova, T-test (n=3/group, ****p<0.001).

Supplementary Figure 3 RNAseq analysis of M2 macrophage treated with lucicebtide. **(A)** Unsupervised clustering of control and lucicebtide treated samples (n=3/group). Heatmap is shown for the 45 genes identified by UC (see **Supplementary Table S3**). Curated M2-linked genes are shown. **(B)** Volcano plot for differentially regulated genes. X axis, log2 fold change between control and lucicebtide-treated cells. Y axis, Log_10_ of Student-Test p-values for each gene. Horizontal dotted line indicates the p=0.05 threshold. Vertical dotted lines represent 1.5 fold changes in both directions. Ctrl up, red indicate genes upregulated in Control versus Lucicebtide. Lucicebtide up (blue) represent genes upregulated in lucicebtide treated cells versus control. List of genes and statistics in **Supplementary Table S4**. **(C)** Expression levels for previously characterized C/EBPβ targets in control and lucicebtide-treated samples (n=3/group). Statistics, 2-way Anova Student T-test with Sidak correction; ns, not significant; *,p<0.05;****p<0.0001). **(D)** GSEA plots for two representative gene sets enriched in control sample (left) and two enriched in lucicebtide-treated conditions. C, Control; L, Lucicebtide. Gene sets and related statistics for the top 20 sets enriched in either class are listed in **Supplementary Table S5**.

Supplementary Figure 4 CEBPB-bound signature identifies inferior prognosis in HR+ BC. **(A)** Distribution of TCGA HR-positive BC samples according to the CEBPB-bound signature in top quartile (Top25, blue, n=149) and lower three quartiles (Lower75, red, n=445). Horizontal lines indicate score averages. **(B)** Survival curves for the Lower75 and Top25 sets (Median Survivals; Top25,11.69 yrs; Bottom75,10.85 yrs). Statistics indicate log-rank test.

Supplementary Figure 5 Weights for mice transplanted with 4T1 cells and treated with vehicle (gray) or with lucicebtide at the indicated dosing (green, 10mg/kg; blue 25 mg/kg; red, 50mg/kg) and monitored at the indicated times.

Supplementary Figure 6
**(A, C)** Distributions of Xcell Immune Infiltrate scores for Macrophage (M), M1-type (M1) and M2-type (M2) cells for ovarian cancer **(A)** and GBM **(C)**. For zero values, a marginal value of 1E-20 was added to allow log_10_ visualization. Horizontal bars represent median values. **(B, D)** Relative frequencies for the indicated categories classified in High (H, red bars) and Low (L, blue) according to the infiltrate median level and in Lower75 or Top25 based on CEBPB transcript level for ovarian cancer **(B)** and GBM **(D)**. Statistics, Fisher T-test p-values. The observed frequencies used for contingency tests are reported in **Supplementary Table S6**.

Supplementary Figure 7
**(A)** Tumor volumes for CT26 transplanted mice measured at the indicated time points and treated with the indicated regimens (grey, vehicle; green, lucicebtide 10mg/kg; blue 25 mg/kg; red, 50mg/kg). Statistics indicate 1-way ANOVA (**,p<0.01; *,p<0.05; n=5/group) for volumes at Day 25. **(B)** Tumor volumes of mice transplanted with CT26 cells and treated with vehicle (gray) or with the indicated treatment (lucicebtide, 10 mg/kg, blue; anti-PD-1, 9mg/kg, green; combination, red). Statistics indicate 1-way ANOVA (*,p<0.05; ns, not significant; n=6/group) for volumes at Day 20.

Supplementary Figure 8 CD11b+CD45+ as fractions of singlets from tumors in the indicated cohort. Each cohort is compared to the corresponding anti-CSFR-1 treatment. Statistics indicate 1-way ANOVA (****,p<0.0001; ***,p<0.001; *,p<0.05; n=5/group).

Supplementary Figure 9 Tumor volumes for 4T1R transplanted mice measured at the indicated time points and treated with the indicated regimens. Statistics for the relative growth of tumors are reported in **Figure 4D**.

Supplementary Table 1 List of QPCR primer set used in this study.

Supplementary Table 2 CEBPB-bound gene set list (n=473) used for TCGA-BC signature scoring (**Figures 3A, B**).

Supplementary Table 3 UC Gene list from UC shown in **Supplementary Figure S3A**.

Supplementary Table 4 Differentially expressed genes list and indicated statistics for control *vs* lucicebtide treated M2 cells as reported in **Supplementary Figure S3B**.

Supplementary Table 5 GSEA results for top 20 gene sets enriched in control or lucicebtide-treated classes. Classes are reported in the first column. ES, enrichment score. NES, normalized enrichment score.

Supplementary Table 6 Observed and relative frequencies for classifications according to C/EBPβ transcript level and immune Infiltrate (M, macrophage; M1, M1-type or M2, M2-type macrophage) for ovarian cancer and GBM sets. Statistics report Fisher T-test p-value for the corresponding contingency table.
