# Supplementary figures and images for: The C/EBPβ antagonist peptide lucicebtide (ST101) induces macrophage polarization toward a pro-inflammatory phenotype and enhances anti-tumor immune responses
Source: Front Immunol. 2025 Mar 4;16:1522699. doi: 10.3389/fimmu.2025.1522699 (PMC11913834; doi:10.3389/fimmu.2025.1522699)

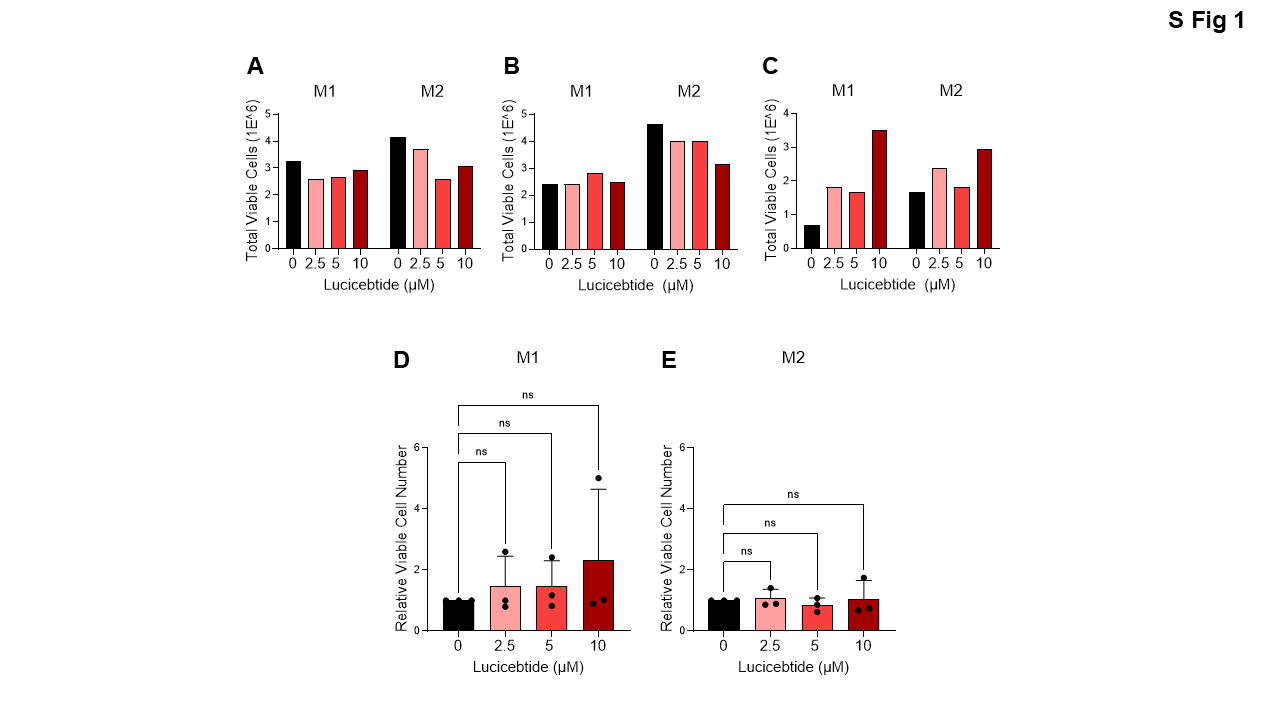

Supplement: Supplementary Figure 1 — Lucicebtide does not reduce total cell numbers in M1 or M2 cultures. (A-C) Total viable cells reported for three independent hPBMC-derived M1 or M2 cultures exposed for 10 days to the indicated lucicebtide concentrations or left untreated. Averages of the relative viable cell number are shown for M1 (D) and M2 (E) lucicebtide-treated cultured from (A-C) ns, not significant by 1-way Anova compared to untreated. [file Image1.tif]

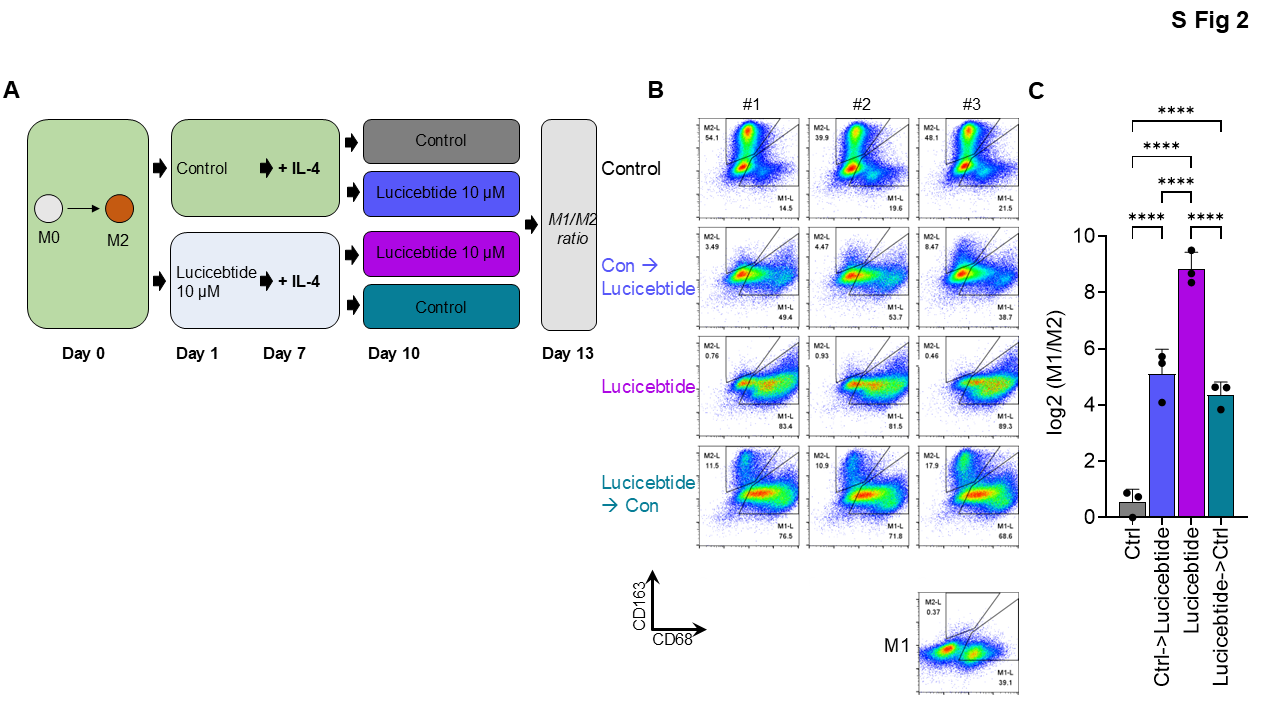

Supplement: Supplementary Figure 2 — Lucicebtide repolarizes established M2 macrophages to the M1 identity. (A) Experimental outline of M2 culture establishment and activation from hPBMCs subject to the following treatments: untreated (gray box); untreated up to day 10 followed by 3-day exposure to lucicebtide 10 µm (blue); treated with lucicebtide 10 µm (violet); treated with lucicebtide 10 µM up to day 10 and switched to untreated media (cyan). All conditions were assessed by flow cytometry on day 13. (B) Flow Cytometry of Day 13 M2 cultures from the experimental condition described in A. M2 populations are manually gated as CD68lowCD163high, M1 populations are CD68highCD163low. An M1 culture is shown for gating control. Statistics indicate percentages of parent population. (C) Log2 M1/M2 Ratio normalized to an untreated control cultures for the indicated condition. Statistics, 1-way Anova, T-test (n=3/group, ****p<0.001). [file Image2.tif]

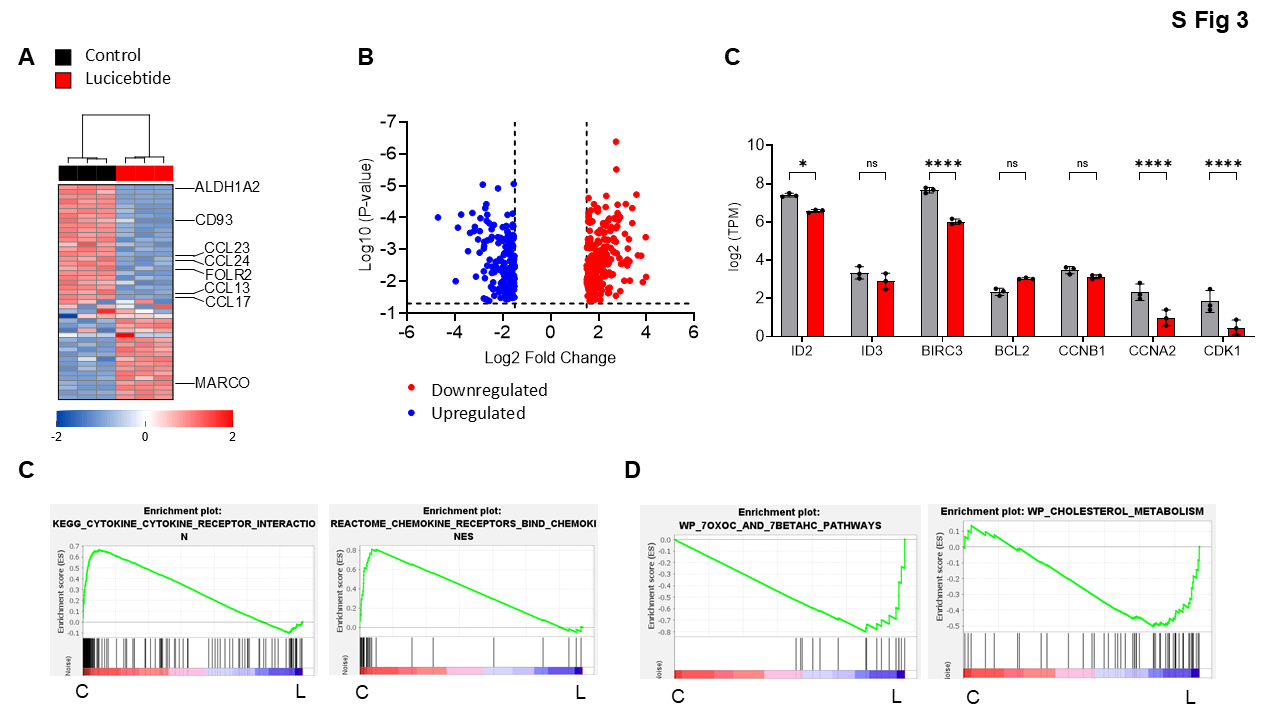

Supplement: Supplementary Figure 3 — RNAseq analysis of M2 macrophage treated with lucicebtide. (A) Unsupervised clustering of control and lucicebtide treated samples (n=3/group). Heatmap is shown for the 45 genes identified by UC (see Supplementary Table S3 ). Curated M2-linked genes are shown. (B) Volcano plot for differentially regulated genes. X axis, log2 fold change between control and lucicebtide-treated cells. Y axis, Log10 of Student-Test p-values for each gene. Horizontal dotted line indicates the p=0.05 threshold. Vertical dotted lines represent 1.5 fold changes in both directions. Ctrl up, red indicate genes upregulated in Control versus Lucicebtide. Lucicebtide up (blue) represent genes upregulated in lucicebtide treated cells versus control. List of genes and statistics in Supplementary Table S4 . (C) Expression levels for previously characterized C/EBPβ targets in control and lucicebtide-treated samples (n=3/group). Statistics, 2-way Anova Student T-test with Sidak correction; ns, not significant; *,p<0.05;****p<0.0001). (D) GSEA plots for two representative gene sets enriched in control sample (left) and two enriched in lucicebtide-treated conditions. C, Control; L, Lucicebtide. Gene sets and related statistics for the top 20 sets enriched in either class are listed in Supplementary Table S5 . [file Image3.tif]

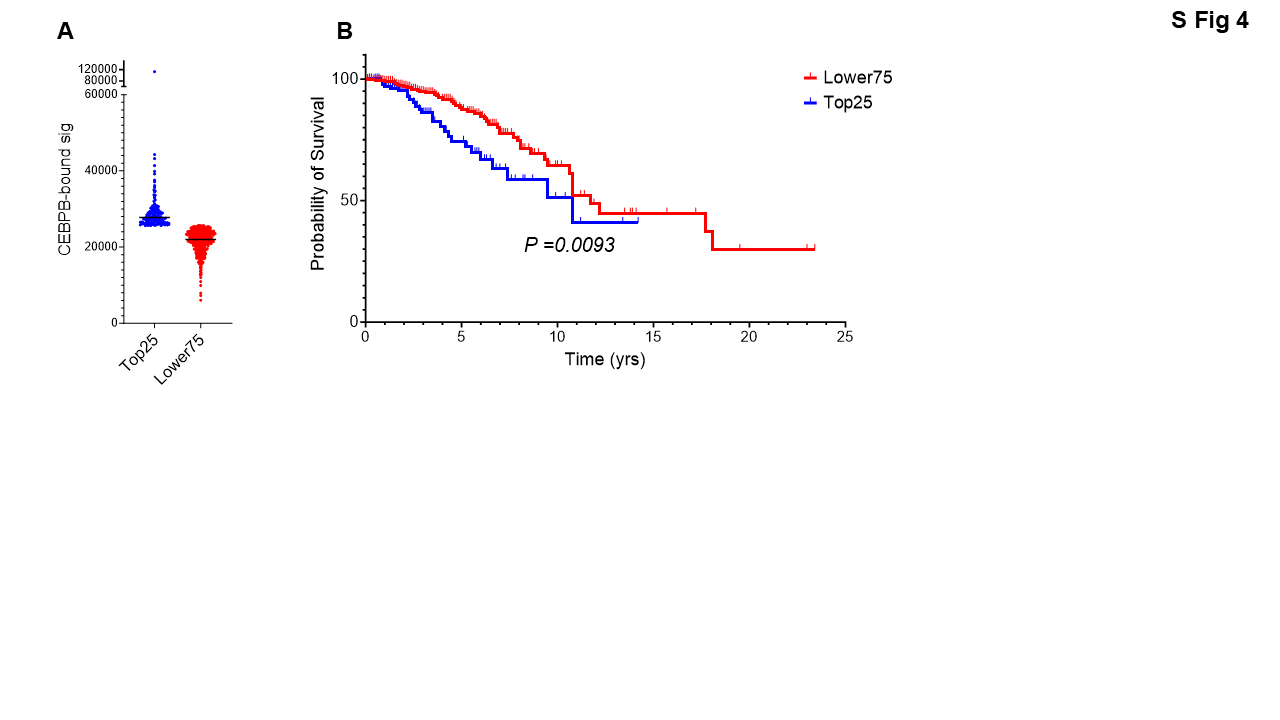

Supplement: Supplementary Figure 4 — CEBPB-bound signature identifies inferior prognosis in HR+ BC. (A) Distribution of TCGA HR-positive BC samples according to the CEBPB-bound signature in top quartile (Top25, blue, n=149) and lower three quartiles (Lower75, red, n=445). Horizontal lines indicate score averages. (B) Survival curves for the Lower75 and Top25 sets (Median Survivals; Top25,11.69 yrs; Bottom75,10.85 yrs). Statistics indicate log-rank test. [file Image4.tif]

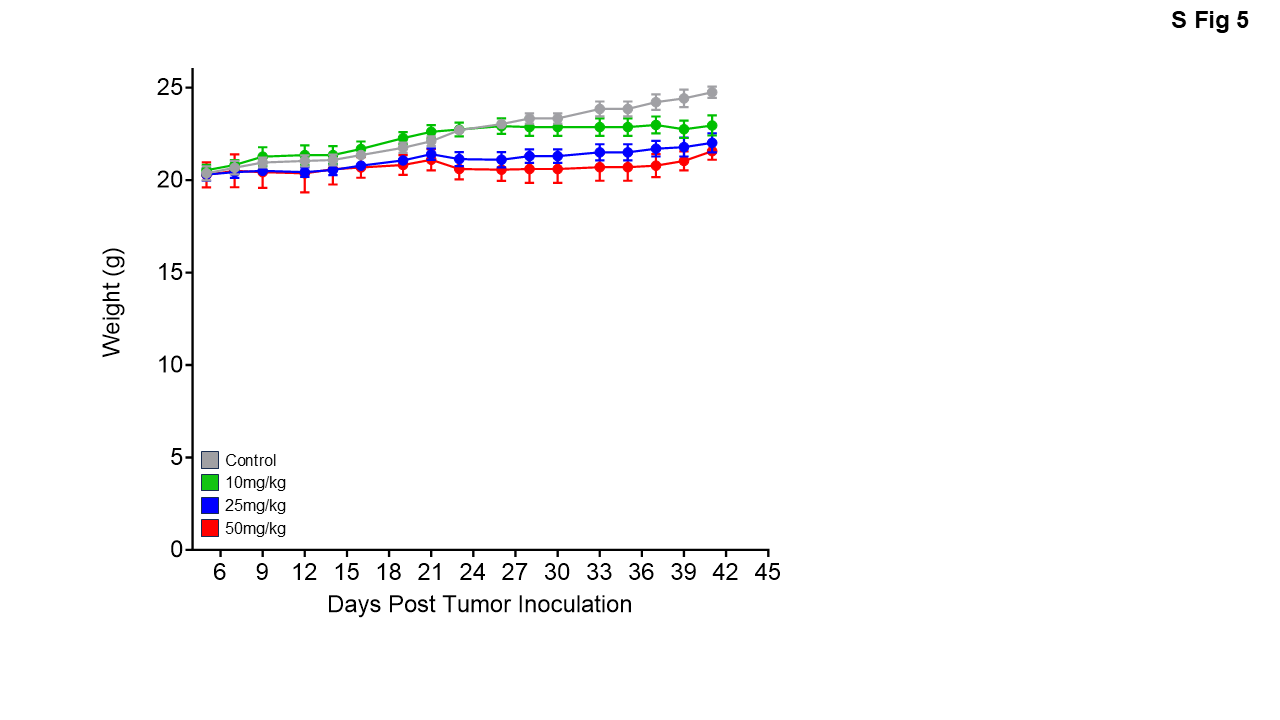

Supplement: Supplementary Figure 5 — Weights for mice transplanted with 4T1 cells and treated with vehicle (gray) or with lucicebtide at the indicated dosing (green, 10mg/kg; blue 25 mg/kg; red, 50mg/kg) and monitored at the indicated times. [file Image5.tif]

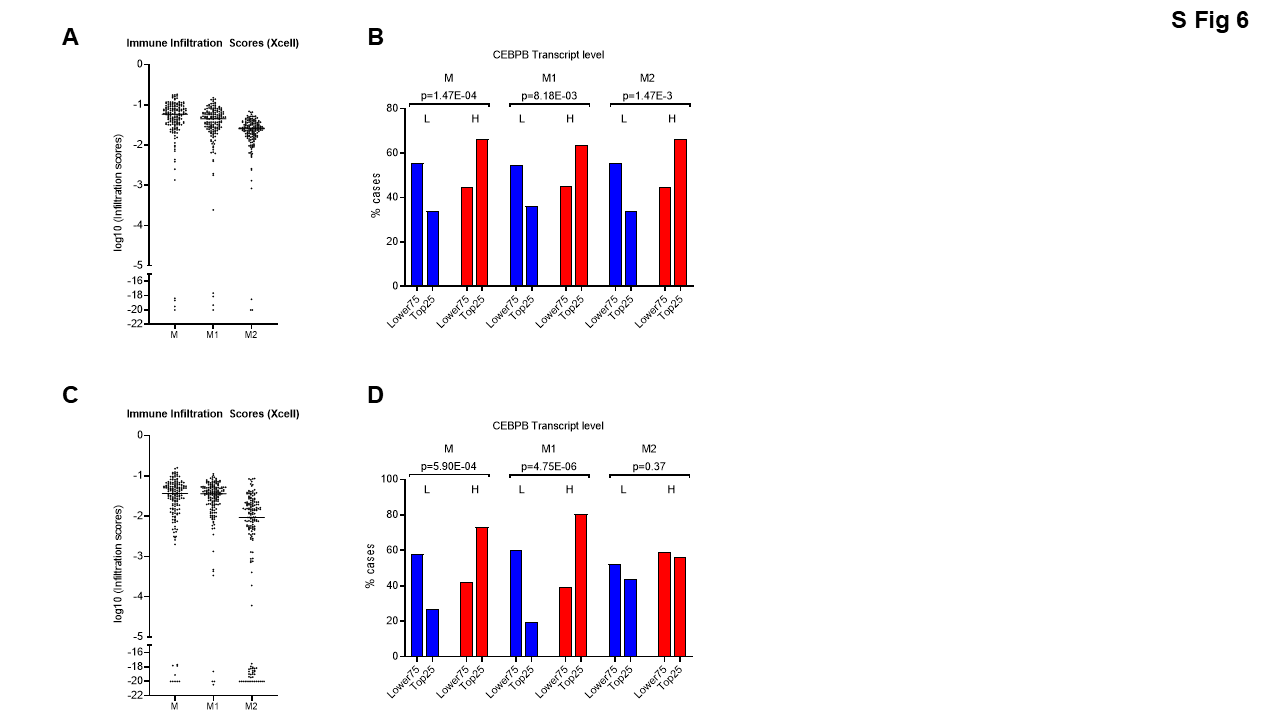

Supplement: Supplementary Figure 6 — (A, C) Distributions of Xcell Immune Infiltrate scores for Macrophage (M), M1-type (M1) and M2-type (M2) cells for ovarian cancer (A) and GBM (C). For zero values, a marginal value of 1E-20 was added to allow log10 visualization. Horizontal bars represent median values. (B, D) Relative frequencies for the indicated categories classified in High (H, red bars) and Low (L, blue) according to the infiltrate median level and in Lower75 or Top25 based on CEBPB transcript level for ovarian cancer (B) and GBM (D). Statistics, Fisher T-test p-values. The observed frequencies used for contingency tests are reported in Supplementary Table S6 . [file Image6.tif]

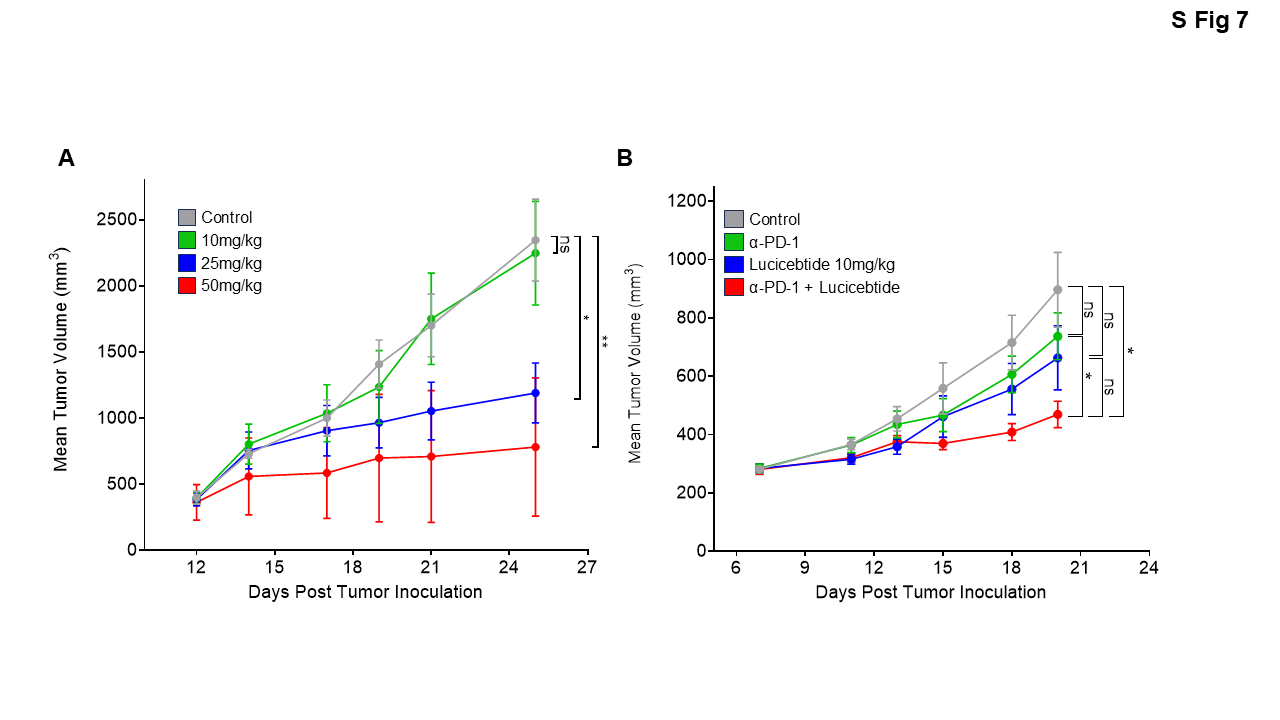

Supplement: Supplementary Figure 7 — (A) Tumor volumes for CT26 transplanted mice measured at the indicated time points and treated with the indicated regimens (grey, vehicle; green, lucicebtide 10mg/kg; blue 25 mg/kg; red, 50mg/kg). Statistics indicate 1-way ANOVA (**,p<0.01; *,p<0.05; n=5/group) for volumes at Day 25. (B) Tumor volumes of mice transplanted with CT26 cells and treated with vehicle (gray) or with the indicated treatment (lucicebtide, 10 mg/kg, blue; anti-PD-1, 9mg/kg, green; combination, red). Statistics indicate 1-way ANOVA (*,p<0.05; ns, not significant; n=6/group) for volumes at Day 20. [file Image7.tif]

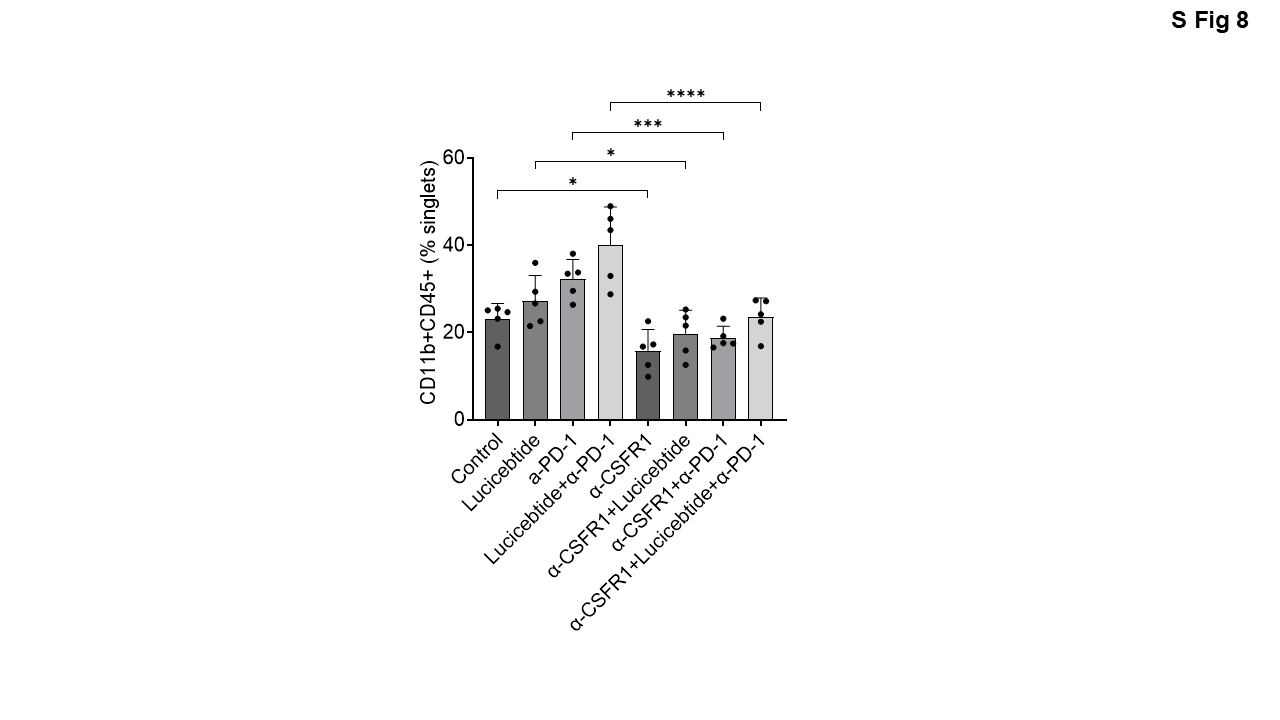

Supplement: Supplementary Figure 8 — CD11b+CD45+ as fractions of singlets from tumors in the indicated cohort. Each cohort is compared to the corresponding anti-CSFR-1 treatment. Statistics indicate 1-way ANOVA (****,p<0.0001; ***,p<0.001; *,p<0.05; n=5/group). [file Image8.tif]

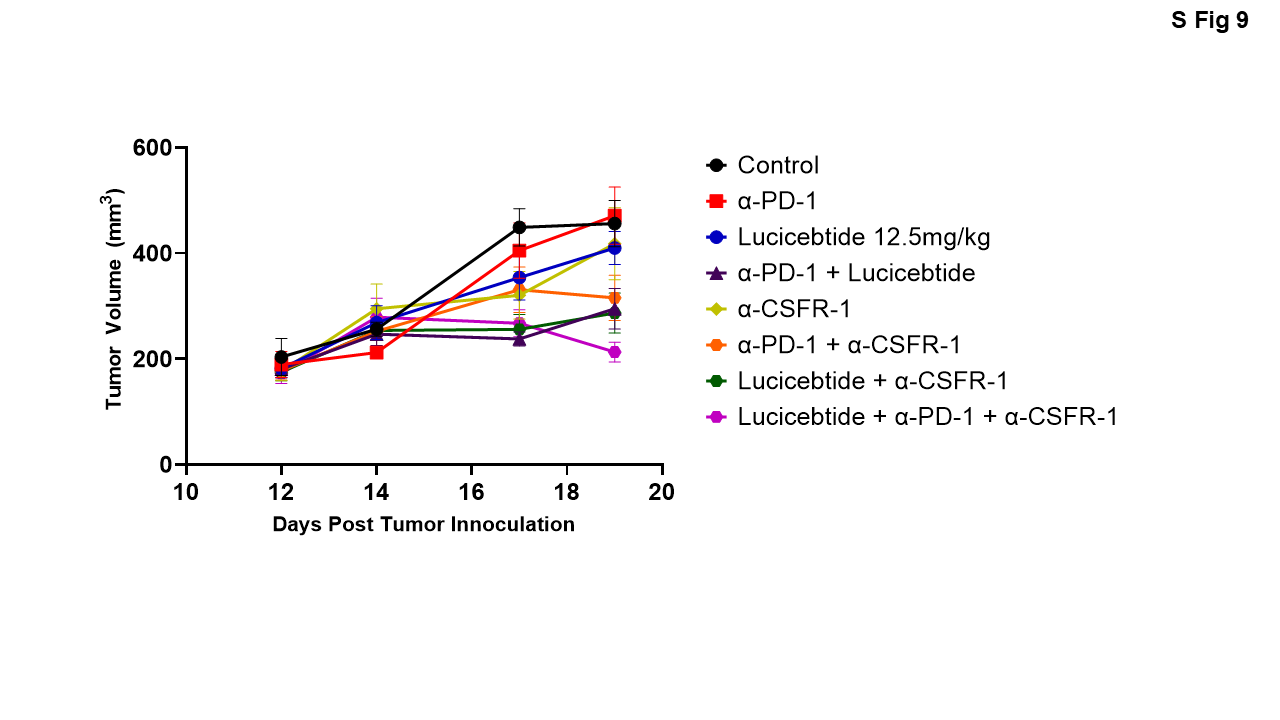

Supplement: Supplementary Figure 9 — Tumor volumes for 4T1R transplanted mice measured at the indicated time points and treated with the indicated regimens. Statistics for the relative growth of tumors are reported in Figure 4D . [file Image9.tif]
